# Impact of water-deficit stress on tritrophic interactions in a wheat-aphid-parasitoid system

**DOI:** 10.1371/journal.pone.0186599

**Published:** 2017-10-20

**Authors:** Syed Suhail Ahmed, Deguang Liu, Jean-Christophe Simon

**Affiliations:** 1 State Key Laboratory of Crop Stress Biology for Arid Areas (Northwest A&F University), Yangling, Shaanxi Province, China; 2 College of Plant Protection, Northwest A&F University, Yangling, Shaanxi Province, China; 3 Institut National de la Recherche Agronomique (INRA), unité mixte de recherche (UMR) 1349, Institut de Génétique, Environnement et Protection des Plantes (IGEPP), Domaine de la Motte, Le Rheu, France; Institut Sophia Agrobiotech, FRANCE

## Abstract

Increasing temperature and CO_2_ concentrations can alter tritrophic interactions in ecosystems, but the impact of increasingly severe drought on such interactions is not well understood. We examined the response of a wheat-aphid-parasitoid system to variation in water-deficit stress levels. Our results showed that arid area clones of the aphid, *Sitobion avenae* (Fabricius), tended to have longer developmental times compared to semiarid and moist area clones, and the development of *S*. *avenae* clones tended to be slower with increasing levels of water-deficit. Body sizes of *S*. *avenae* clones from all areas decreased with increasing water-deficit levels, indicating their declining adaptation potential under drought. Compared to arid area clones, moist area clones of *S*. *avenae* had a higher frequency of backing under severe water stress only, but a higher frequency of kicking under well-watered conditions only, suggesting a water-deficit level dependent pattern of resistance against the parasitoid, *Aphidius gifuensis* (Ashmead). The number of *S*. *avenae* individuals attacked by the parasitoid in 10 min showed a tendency to decrease with increasing water-deficit levels. Clones of *S*. *avenae* tended to have lower parasitism rates under treatments with higher water-deficit levels. The development of the parasitoid tended to be slower under higher levels of water-deficit stress. Thus, the bottom-up effects of water-deficit stressed plants were negative on *S*. *avenae*. However, the top-down effects via parasitoids were compromised by water-deficit, which could favor the growth of aphid populations. Overall, the first trophic level under water-deficit stress was shown to have an indirect and negative impact on the third trophic level parasitoid, suggesting that parasitoids could be increasingly vulnerable in future warming scenarios.

## Introduction

During the past 130 years, an approximate 0.85°C increase in the mean global surface temperature was recorded, and the warming extent could further intensify by 2.5–7.8°C before 2100 [1]. Meanwhile, an increase in extreme weather events (e.g., flooding, storms and drought) is predicted [1]. The warming trend is also evident in China, since significant rises in temperature (1.10C) and increasing numbers of drought spells have been observed in China over the past century of 1908–2007 [2]. Much of the climate change has been attributed to increased CO_2_ concentrations in the atmosphere [1]. The potential effects of increasing temperature and CO_2_ concentrations have been extensively explored, and both factors could have diverse effects (in terms of phenology, physiology, abundance and distribution) on plants, as well as on herbivores [3–4]. Increasing recent attention has been paid to tritrophic interactions in various ecosystems under elevated temperatures and CO_2_ levels [5–6]. For example, it was found that higher temperatures as a result of frequent occurrences of warm springs (a possible consequence of the climate change) could uncouple the phenological synchrony between *Melitaea cinxia* L. and its host plants, as well as between *Cotesia melitaearum* (Wilkinson) (a braconid parasitoid) and its host (*M*. *cinxia*) [7]. Bezemer et al. [8] presented a study of tritrophic interactions under elevated temperature and CO_2_ in a system consisting of four plants, the aphid *Myzus persicae* (Sulzer), and the parasitoid *Aphidius matricariae* (Haliday). In that study, the aphid’s abundances increased under both elevated CO_2_ and temperature, while its parasitism rates showed a tendency to rise with increasing temperatures [8]. Changes in tritrophic interactions may induce destabilization in the population dynamics of certain species by influencing their biology, ecology and evolution, which could ultimately lead towards their extinction [9–10].

Another significant indicator of the global climate change is changing dynamics of drought or water-deficit stress. It was predicted that drought could be more acute, prolonged and frequent in China for the period of 2020–2049 compared to the period of 1971–2000 [11]. Drought may compromise plant defensive strategies and make them more vulnerable to opportunistic herbivores [12]. Drought can also show variable direct impacts on herbivores depending on its mode (i.e., pulsed or continuous) and intensity [13–14]. Although the impact of drought on herbivores has been controversial [15–17], quite a few aphid species, such as the Russian wheat aphid [*Diuraphis noxia* (Kurdjumov)], corn leaf aphid [*Rhopalosiphum maidis* (Fitch)], greenbug [*Schizaphis graminum* (Rondani)], and cabbage aphid (*Brevicoryne brassicae* L.), manifested positive responses under water stressful conditions [18–21]. In addition, decreases in precipitation may affect parasitoids more than their host herbivores because of the cascading effects [22]. Thus, water-deficit stress can contribute to alteration in tritrophic interactions in agricultural ecosystems in the context of global warming [23–25]. However, studies in this respect have been rare and the mechanisms underlying the abovementioned interactions are not well understood.

In northwestern China, the warming trend is particularly evident [2], and this provides an excellent scenario for addressing the abovementioned issues. The wheat aphid, *Sitobion avenae* (Fabricius), has been shown to cause increasing damage to wheat production in this part of China [26]. This aphid has the ability to adapt rapidly to various unfavorable environments including water-deficit stress [27–32]. The endoparasitoid, *Aphidius gifuensis* (Ashmead), is one of the most important natural control agent for *S*. *avenae* on wheat [33–34]. So, we hypothesize that *S*. *avenae* clones from moist, semiarid and arid areas of northwestern China should have differential performance on water-deficit stressed host plants, and this in turn would affect the fitness of the parasitoid (*A*. *gifuensis*) under different water-stress treatments. The objectives of our study were to address the tritrophic interactions in the wheat-aphid-parasitoid system. Specifically, we aim to: 1) determine how water-stressed wheat plants affect the development and body size of *S*. *avenae* clones from moist, semiarid and arid areas; 2) examine how *S*. *avenae* clones of different areas respond to the attack of the parasitoid (*A*. *gifuensis*) under water-deficit conditions; and 3) evaluate the performance of *A*. *gifuensis* on all developmental stages of *S*. *avenae* under three water-stress treatments.

## Materials and methods

### Colony establishment

**Fig 1:**
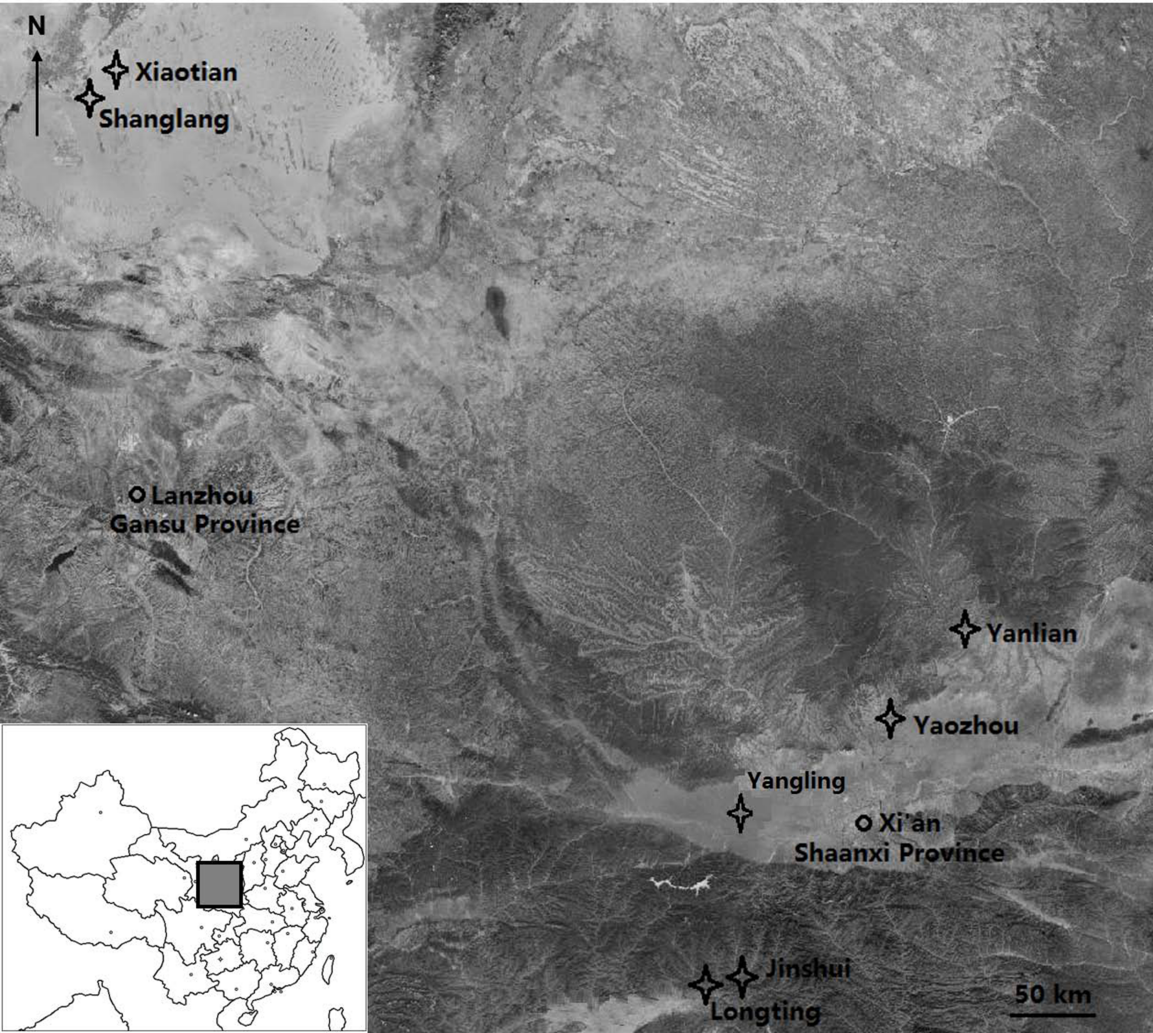
Sampling locations of *Sitobion avenae* and *Aphidius gifuensis* (moist area: Longting Town of Yangxian Co., 33°12'43"N, 107°38'30"E; Jinshui Town of Yangxian Co., 33°16'20" N, 107°47'45" E; semiarid area: Yanlian Co., 35°41’29” N, 109°16’13” E; Yaozhou Co., 34° 53’38” N, 108°58’18” E; arid area: Shanglang Town of Minqin Co., 38°35'48" N, 103°06'17" E; Xiaotian Town of Minqin Co., 38°36'40" N, 103°07'18" E; *A*. *gifuensis* larvae sampled in Yangling, 34°16’56” N, 108°04’28” E).

Following previous studies [26–27], the areas with a mean annual precipitation up to 200 mm or less, 200–400 mm, and 800 mm or more were considered to be arid, semiarid, and moist, respectively. *Sitobion avenae* sample collections were made at two locations in each area from April to June 2015 (Fig 1). At least 20 apterous adults were sampled per location. Individuals were collected from wheat plants with a minimum distance of 10 m in order to minimize the probability of sampling identical clones [32]. The aphid samples were genotyped using six microsatellite loci (Sa4Σ, Sm10, Sm12, Sm17, S5L and S17b) [28,35–36]. Six distinct genotypes from each source area were randomly selected for use in the following bioassays. All the selected genotypes were used to establish segregated stock colonies in the laboratory. These genotypes were reared on winter wheat seedlings (*Triticum aestivum* cv. Aikang 58) planted in plastic pots (7 cm in diameter). These pots contained a mixture of turfy soil, vermiculite, and perlite (4:3:1, v/v/v), and were well covered with transparent plastic tubes (7cm in diameter, 20 cm in height), which had a net on top (60 mesh) for ventilation. A small plastic pan was put at the base of each pot for providing suitable amount of water when needed.

Mummies of *S*. *avenae* that contained developing *A*. *gifuensis* larvae were sampled from local wheat fields in Yangling, Shaanxi Province (Fig 1) in June 2015. This location belongs to semiarid areas according to previous studies [26–27]. No particular permits have been required for insect collecting activities at all the sites mentioned above, and our target insects are not endangered or protected. Cultured stocks of *S*. *avenae* and *A*. *gifuensis* were kept and maintained in a climate room, controlled at a constant temperature of 21 ± 2°C, a relative humidity of 65 ± 5%, and a photoperiod of 16L: 8D, for 2 or 3 generations before experiments. This could minimize the confounding maternal effect of different source environments [31]. To obtain synchronous cohorts of all developmental stages (same age and size), apterous *S*. *avenae* adults from stock colonies were shifted to experimental seedlings, and the offspring produced in 6 h were then kept separately for later use in experiments.

### Water level treatments

The experiments were carried out under continuous water-deficit stress. We established three treatments: well-watered, intermediately water-stressed, and severely water-stressed. These water treatments were conducted as detailed previously in [26]. Specifically, we applied 10, 7, and 5 ml of water every 3 d for wheat seedlings (*T*. *aestivum cv*. Aikang 58) with 35 g (dry weight) of growing media (i.e., the abovementioned mixture of turfy soil, vermiculite, and perlite) under well-watered, intermediately stressed, and severely stressed treatments, respectively. The resulting intermediate and severe water-deficit stress in the latter two treatments (according to [37]) was monitored by measuring soil water potential using a tensiometer (TEN30, Top Instrument, Hangzhou, China). The soil water potential in well-watered, intermediately stressed and severely stressed treatments was kept at a range of -0.02 to -0.01MPa, -0.035 to -0.02MPa and lower than -0.045MPa, respectively. Water potential was also measured in wheat leaves using the Chardakov method [38]. The corresponding water potential of wheat leaves in the three treatments was kept at a range of 0 to -0.2 Mpa, -0.2 to -0.6 MPa and -0.6 to -0.8 Mpa, respectively [27]. We kept the desirable water conditions in pots of wheat seedlings by measuring soil water potential and weighing the plants with the growing media twice a week.

### Developmental time and body size of *S*. *avenae* clones

Data on developmental time and body size were collected as described previously in [31–32]. Briefly, 7–8 d old seedlings (the same wheat cultivar mentioned above) were inoculated with aphid individuals (one per plant) from rearing plants. Each seedling with a single aphid individual was covered with the abovementioned plastic tubes, and kept in a growth chamber with the following conditions: 20 ± 1°C (temperature), 65 ± 5% (relative humidity), and 16L: 8D (photoperiod). Six clones were randomly selected from aphid colonies of each area (i.e., arid, semi-arid and moist) for use in the bioassays, and the experiment was replicated three to four times for each clone. We monitored aphid individuals in the bioassays twice daily from birth until the emergence of adults, and recorded the timing of molts. Seven to nine days after the initiation of the experiment, newly emerged apterous adults of *S*. *avenae* clones from each area were used to measure the body length (μm) from the front of head to the end of abdomen (cornicles were excluded). The measurement was conducted under a dissecting microscope (Motic K-400L, Motic China Group Co. Ltd., Xiamen, China) using the software of Motic Images Advanced (version 3.2).

### Preference of *A*. *gifuensis*

For this test, mated *A*. *gifuensis* females (1–2 d old) were transferred to pots of wheat seedlings. Each pot had a 7–8 d old wheat plant with well settled 20 aphids of each clone. A period of t10–15 min was needed for the aphids to settle on the plant. Each developmental stage of *S*. *avenae* was tested separately. We chose to use plastic pots containing wheat seedlings and aphid individuals for the experiment instead of petri dishes in order to mimic natural conditions. After its introduction into the cage, *A*. *gifuensis* was allowed to acclimate to the cage environment for 5 min. After that, we recorded the frequency of contacts between female *A*. *gifuensis* and the plant (i.e., the landing of *A*. *gifuensis* on the plant surface) for 10 min, and the number of aphid individuals attacked by *A*. *gifuensis* in the same time span. This experiment was replicated four times for each clone of the aphid.

### Parasitoid attack and aphid resistance

Using similar settings in the abovementioned test, the number of attacks on single adult aphid (apterous) individuals by *A*. *gifuensis* in 30 min was recorded. In this test, one mated *A*. *gifuensis* female (1–2 d old) was released onto a 7–8 d old wheat seedling caged with one newly emerged apterous aphid adult, and allowed to acclimate for 5 min. The number of times for a female *A*. *gifuensis*’s probing with its ovipositor was considered as the number of attacks on the aphid. For the test on aphid resistance behaviors, the system was monitored for at least 10 min after allowing the parasitoid to acclimate for 5 min. It took the parasitoid at least 10 min to find the aphid on a wheat plant. Upon the encounter between them, the aphid chose to back, kick or drop from the plant. We then recorded the aphid resistance behavior for the first encounter between the parasitoid and the aphid. Both experiments were replicated four times for each aphid clone.

### Parasitism

This experiment was designed to examine the potential parasitism by *A*. *gifuensis* against all aphid developmental stages of *S*. *avenae* from areas of different drought levels. To ensure the occurrence of mating, newly emerged pairs (1 male and 1 female) of *A*. *gifuensis* were transferred to a gelatin capsule with 10% honey solution, and kept there for 24 h [39]. On the other hand, 20 aphid nymphs or adults of the same age were introduced to the experimental wheat seedling (one or two-leaf plant) which was planted in a plastic pot as described above. Upon the settlement of aphid individuals on wheat seedlings, a mated pair of *A*. *gifuensis* was introduced into the plastic cage, and kept there for 6 h. After that, aphid individuals were maintained therein for up to 10–15 d (from the day of parasitoid exposure) under the same environmental conditions. They were examined carefully every 12 h from day 7, and mummified aphid individuals were isolated from plants and placed in petri dishes. Numbers of mummies and the dates of emergence were recorded. All aphid developmental stages were tested, and the test was replicated four times for each aphid clone.

### Statistical analyses

Nested analyses of variance (nested ANOVAs) in SAS [40] were used to identify the effects of source areas, water treatments, and their interactions for the following parameters: the developmental time of 1^st^ to 4^th^ instar nymphs (DT1 to DT4) of *S*. *avenae*, the developmental time of the entire nymphal stage (DT5) for *S*. *avenae*, body size of *S*. *avenae*, developmental time of *A*. *gifuensis*, frequency of *A*. *gifuensis* attacks, number of *S*. *avenae* individuals attacked, and parasitism rates. If overall variation in ANOVAs was significant, mean separations were conducted using Tukey tests at α = 0.05. We used log-transformation of data when necessary to meet the requirements of normality and homoscedasticity in ANOVAs. Aphid resistance behaviors (i.e., backing, kicking and dropping), as well as the plant contact behavior of *A*. *gifuensis*, were analyzed with logistical regressions using the PROC LOGISTIC procedure in SAS [40].

## Results

### Developmental time and body size of *S*. *avenae*

Clones of *S*. *avenae* from arid areas tended to have longer developmental times compared to those from semiarid or moist areas (Table 1). Under the well-watered treatment, DT2 for arid area clones was longer than that for semiarid or moist area clones (*F* = 8.72; *df* = 2, 153; *P* < 0.001). Under well-watered and intermediately stressed conditions, arid area clones showed longer DT3 (*F* = 12.18; *df* = 2, 153; *P* < 0.001) and DT5 (*F* = 14.64; *df* = 2, 153; *P* < 0.001) than semiarid or moist area clones.

**Table 1:** Developmental times (SE) of four instars and the entire nymphal stage for *Sitobion avenae* clones from arid, semiarid and moist areas under three water treatments.

Source	Treatment	DT1	DT2	DT3	DT4	DT5
Arid area	Well-watered	1.9AB (0.1)	1.8AB (0.1)	1.9ABC (0.2)	2.1AB (0.1)	7.8AB (0.2)
	Intermediately stressed	2.0AB (0.2)	2.0A (0.2)	2.1AB (0.2)	2.1AB (0.1)	8.1A (0.1)
	Severely stressed	1.7B (0.1)	2.1A (0.1)	2.3A (0.2)	2.0ABC (0.1)	8.1A (0.1)
Semiarid area	Well-watered	2.0AB (0.1)	1.3C (0.1)	1.4D (0.1)	2.3A (0.1)	7.0C (0.2)
	Intermediately stressed	2.2AB (0.1)	1.8AB (0.1)	1.6CD (0.2)	1.7C (0.1)	7.3BC (0.2)
	Severely stressed	1.9AB (0.2)	1.8AB (0.1)	2.1AB (0.1)	1.9BC (0.2)	7.7AB (0.2)
Moist area	Well-watered	2.0AB (0.1)	1.3C (0.1)	1.4D (0.1)	2.1AB (0.1)	6.8C (0.2)
	Intermediately stressed	1.8AB (0.1)	1.8AB (0.1)	1.4D (0.1)	1.9BC (0.1)	7.0C (0.2)
	Severely stressed	2.3A (0.1)	1.6BC (0.1)	1.8BCD (0.1)	2.2AB (0.1)	7.9AB (0.3)

Note: DT1 to DT4, developmental time of 1^st^ to 4^th^ instar; DT5, developmental time of the entire nymphal stage; n = 18; different letters after data within a column indicate significant differences among treatments at the *P* < 0.05 level, nested ANOVA followed by Tukey tests.

The developmental times for *S*. *avenae* clones tended to rise with increasing water-deficit levels in water treatments. This pattern was most evident in DT2 (*F* = 7.45; *df* = 2, 153; *P* < 0.001), DT3 (*F* = 9.02; *df* = 2, 153; *P* < 0.001) and DT5 (*F* = 9.35; *df* = 2, 153; *P* < 0.001) for semiarid area clones. This pattern was also identified in DT2 and DT5 for moist area clones. However, there were no significant differences in DT1-DT5 among water treatments for arid area clones.

**Fig 2:**
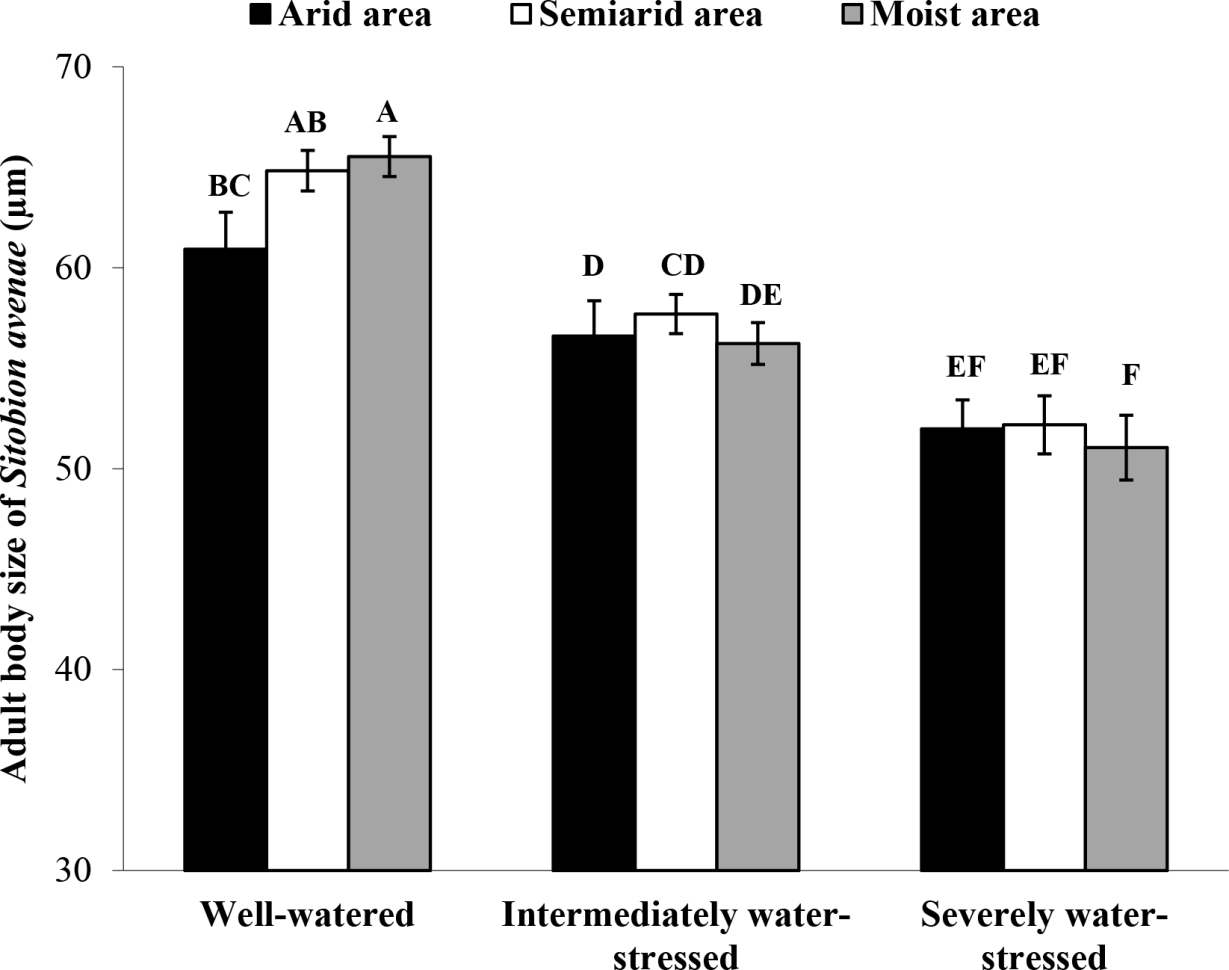
Comparison of body sizes of newly emerged adults of *Sitobion avenae* clones from arid, semiarid and moist areas under three water treatments (n = 18; different letters on bars indicate significant differences among treatments at the *P* < 0.05 level, nested ANOVA followed by Tukey tests).

No significant differences in adult body size of *S*. *avenae* were identified among source areas under all water treatments but the well-watered treatment, where the body size of moist area clones was higher than that of arid area clones (Fig 2; *F* = 17.93; *df* = 2, 153; *P* < 0.001). For each source area, body size of *S*. *avenae* decreased with increasing levels of water-deficit in water treatments (*F* = 57.02; *df* = 2, 153; *P* < 0.001).

### Plant contact behavior of *A*. *gifuensis*

**Fig 3:**
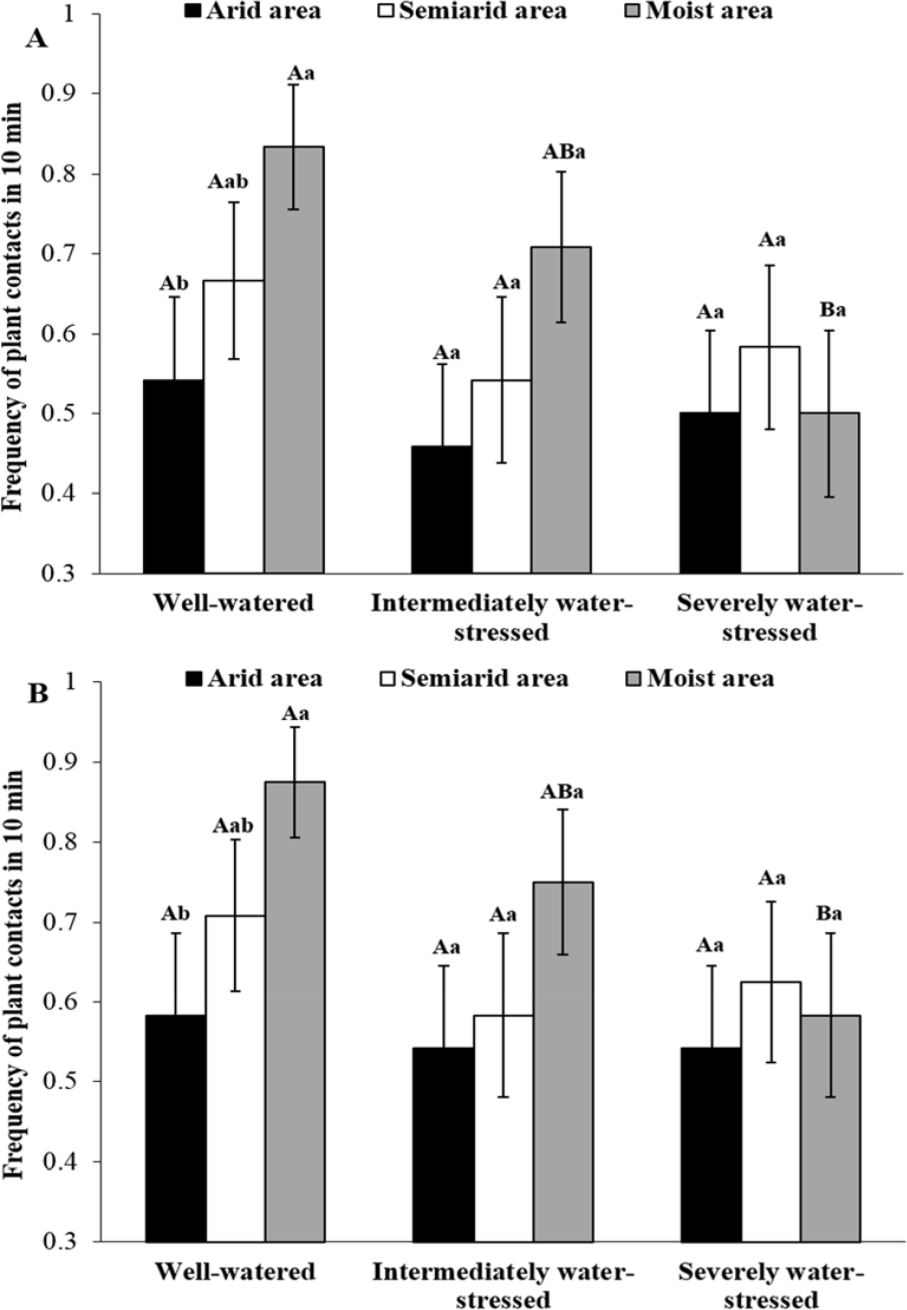
Mean frequencies (/10 min) of *Aphidius gifuensis* contacts with plants carrying 4^th^ intar (A) and adult (B) individuals of *Sitobion avenae* under three water treatments (n = 24; different uppercase and lowercase letters on bars indicate significant differences among water treatments within an area and among areas within a water treatment, respectively; logistic regression, *P* < 0.05).

Under the well-watered treatment, moist area clones showed higher frequency of *A*. *gifuensis* plant contacts than arid area clones when using the 4^th^ nymphal instar of *S*. *avenae* (Fig 3A; logistic regression: χ^2^ = 4.45, *P* < 0.05). Moist area clones had a higher frequency of *A*. *gifuensis* plant contacts under the well-watered treatment than under the severely stressed treatment (logistic regression: χ^2^ = 5.55, *P* < 0.05). In all the other cases, source area and water stress did not influence the frequency of plant contact behavior for the parasitoid. The same pattern in the *A*. *gifuensis* plant contact behavior was found when plants were inoculated with *S*. *avenae* adults instead of 4^th^ instar nymphs (Fig 3B).

### Number of *A*. *gifuensis* attacks and aphid individuals attacked

**Fig 4:**
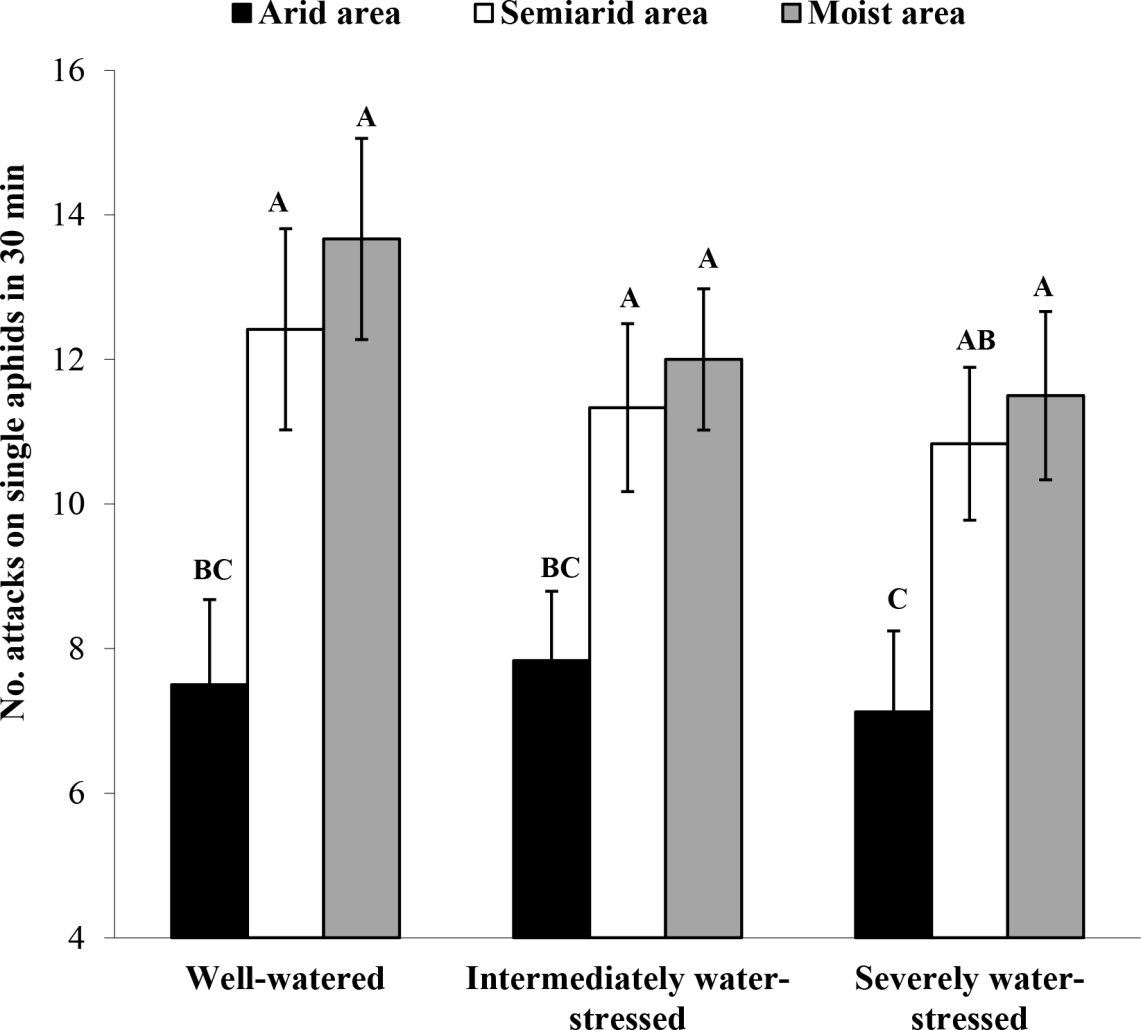
Number of *Aphidius gifuensis* attacks in 30 min on apterous adults of *Sitobion avenae* clones from arid, semiarid and moist areas under three water treatments (n = 24; different letters on bars indicate significant differences among treatments at the *P* < 0.05 level, nested ANOVA followed by Tukey tests).

The frequency of *A*. *gifuensis* attacks (i.e., probes /30 min) on arid area clones of *S*. *avenae* was significantly lower than that on semiarid or moist area clones under all the three water treatments (Fig 4; *F* = 15.14; *df* = 2, 207; *P* < 0.001). There were no significant differences among the three water treatments in the number of *A*. *gifuensis* attacks on *S*. *avenae* clones from any area.

No significant differences in the numbers of *S*. *avenae* individuals attacked by *A*. *gifuensis* in 10 min were found among source areas or among water treatments except 3^rd^ nymphal instar to the adult stage of moist area clones, in which the number of attacks was higher under well-watered conditions compared to severe water stress, with intermediate values for intermediate water stress (Table 2; 3^rd^ instar: *F* = 3.80; df = 2, 207; *P* < 0.05; 4^th^ instar: *F* = 5.47; df = 2, 207; *P* < 0.01; adult: *F* = 7.07; df = 2, 207; *P* < 0.01).

**Table 2:** Numbers (SE) of aphid individuals attacked in 10 minutes by *Aphidius gifuensis* for different developmental stages of *Sitobion avenae* under three water treatments.

Source	Treatment	1^st^ instar	2^nd^ instar	3^rd^ instar	4^th^ instar	Adult
Arid area	Well-watered	1.2A (0.3)	1.6A (0.4)	1.7ABC (0.4)	2.0BCD (0.4)	2.3BCD (0.5)
	Intermediately stressed	1.0A (0.3)	1.1A (0.4)	1.5BC (0.4)	1.5D (0.4)	1.6D (0.3)
	Severely stressed	1.1A (0.3)	1.5A (0.4)	1.4BC (0.4)	1.7CD (0.4)	1.8CD (0.4)
Semiarid area	Well-watered	1.4A (0.3)	1.7A (0.4)	2.4AB (0.4)	2.8ABC (0.4)	3.4AB (0.5)
	Intermediately stressed	1.0A (0.3)	1.4A (0.4)	1.5BC (0.4)	1.8CD (0.4)	2.0CD (0.4)
	Severely stressed	1.2A (0.3)	1.5A (0.4)	2.1ABC (0.4)	2.3BCD (0.4)	2.4BCD (0.4)
Moist area	Well-watered	1.1A (0.3)	1.9A (0.4)	2.7A (0.4)	3.7A (0.4)	4.2A (0.5)
	Intermediately stressed	1.0A (0.3)	1.4A (0.4)	1.4BC (0.4)	3.0AB (0.5)	2.9BC (0.4)
	Severely stressed	0.8A (0.3)	0.9A (0.3)	1.2C (0.4)	1.8CD (0.4)	2.3BCD (0.4)

Note: n = 24; different letters after data within a column indicate significant differences among treatments at the *P* < 0.05 level, nested ANOVA followed by Tukey tests.

### Resistance behaviors of *S*. *avenae* confronting a parasitoid

**Fig 5:**
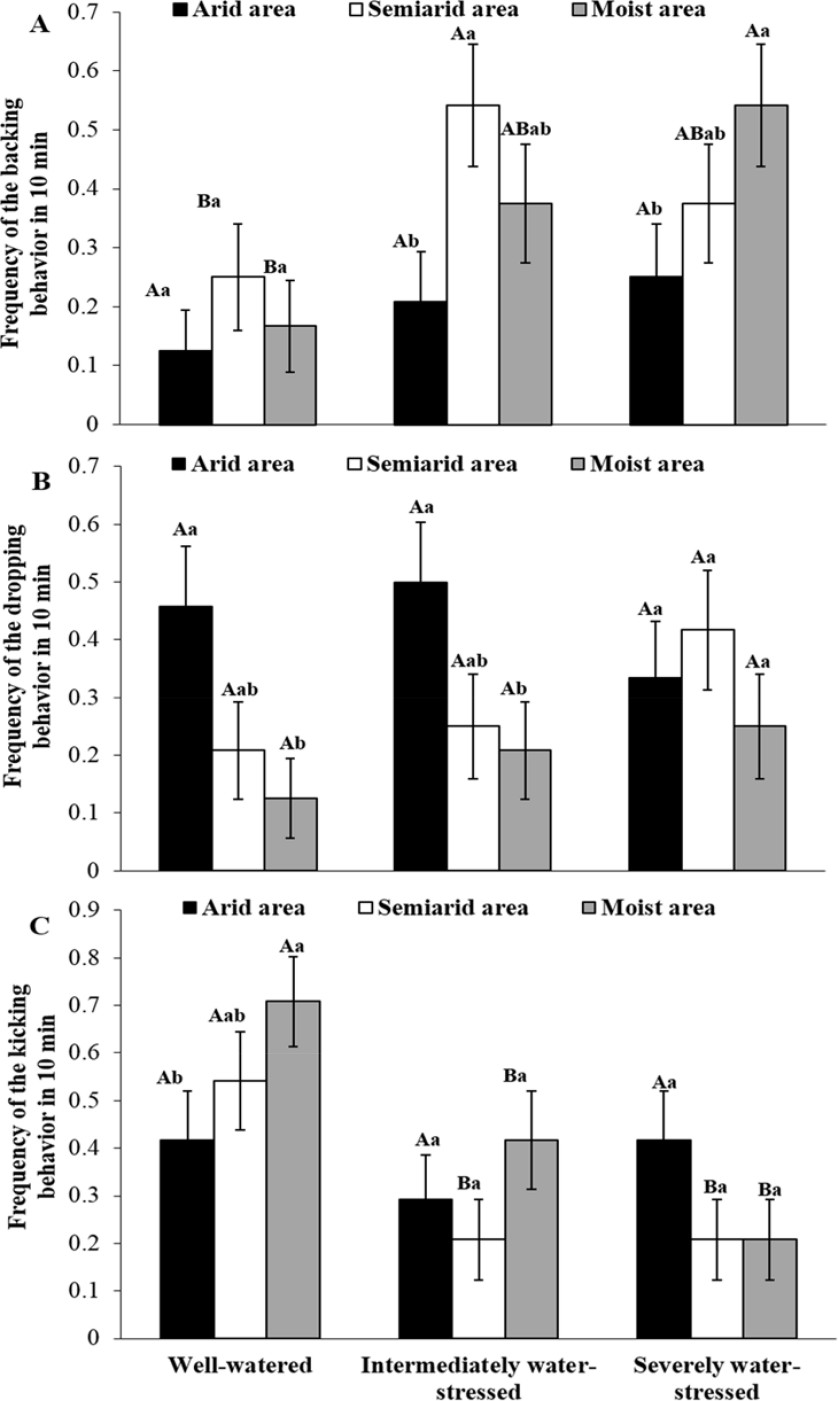
Mean frequencies of behavioral resistance against *Aphidius gifuensis* for adults of *Sitobion avenae* clones from arid, semiarid and moist areas under three water treatments (A, backing; B, dropping; C, kicking; n = 24; different uppercase and lowercase letters on bars indicate significant differences among water treatments within an area and among areas within a water treatment, respectively; logistic regression, *P* < 0.05).

The frequency of backing for arid area clones of *S*. *avenae* was lower than that for semiarid area clones under the intermediately stressed treatment (Fig 5; logistic regression: χ^2^ = 5.37, *P* < 0.05), and it was also lower than that for moist area clones under the severely stressed treatment (logistic regression: χ^2^ = 4.11, *P* < 0.05). Semiarid (intermediate water stress, logistic regression: χ^2^ = 4.11, *P* < 0.05) and moist (severe water stress, logistic regression: χ^2^ = 6.75, *P* < 0.01) area clones backed more often under water-stressed conditions than under well-watered conditions.

Arid area clones dropped more often than moist area clones under all water treatments (well-watered: logistic regression, χ^2^ = 5.46, *P* < 0.05; intermediate stress: logistic regression, χ^2^ = 4.25, *P* < 0.05) except severe water stress for which *S*. *avenae* clones of the three areas showed similar frequency of dropping. No significant differences in frequency of dropping for *S*. *avenae* clones from any area were found among water treatments.

Under the well-watered treatment, arid area clones of *S*. *avenae* had a lower frequency of kicking than moist area clones (logistic regression: χ^2^ = 4.01, *P* < 0.05). Semiarid area clones had a higher frequency of kicking under the well-watered treatment than under the other two water treatments (intermediately stressed: logistic regression, χ^2^ = 5.37, *P* < 0.05; severely stressed: logistic regression, χ^2^ = 5.36, *P* < 0.05). Moist area clones were also found to have a higher frequency of kicking under well-watered conditions than under the other two water treatments (intermediately stressed: logistic regression, χ^2^ = 4.01, *P* < 0.05; severely stressed: logistic regression, χ^2^ = 10.87, *P* < 0.001).

Overall, under well-watered conditions, the percentage of backing, dropping and kicking for *S*. *avenae* clones (three areas combined) was 18.1%, 26.4% and 55.5%, respectively. The respective percentage of backing, dropping and kicking was 37.5%, 31.9% and 30.6% under intermediate water stress, and it was 38.9%, 33.3% and 27.8% under severe water stress.

### Parasitism

Arid area clones tended to have lower parasitism rates in comparison to *S*. *avenae* clones of the other two areas. Under all three water treatments, 1^st^ instar nymphs of arid area clones showed a lower parasitism rate than those of semiarid or moist area clones (Table 3; *F* = 59.06; df = 2, 207; *P* < 0.001); a similar pattern was found for 2^nd^ instar nymphs (*F* = 17.10; df = 2, 207; *P* < 0.001); for 3^rd^ instar nymphs, arid area clones showed a lower parasitism rate than moist area clones under well-watered or severely stressed conditions (*F* = 8.13; df = 2, 207; *P* < 0.001). Adults of *S*. *avenae* from areas of higher drought levels tended to have lower parasitism rates under all three water treatments (*F* = 31.19; df = 2, 207; *P* < 0.001).

**Table 3:** Parasitism rates (SE) of *Aphidius gifuensis* on all developmental stages of *Sitobion avenae* clones from arid, semiarid and moist areas under three water treatments.

Source	Treatment	1^st^ instar	2^nd^ instar	3^rd^ instar	4^th^ instar	Adult
Arid area	Well-watered	46.5CD (0.6)	62.2CD (0.8)	64.1CD (1.2)	46.2BC (0.8)	42.2B (0.7)
	Intermediately stressed	43.2E (0.7)	59.2E (0.8)	64.2BCD (0.7)	42.9D (0.6)	35.8D (0.7)
	Severely stressed	39.4F (0.7)	55.0F (1.2)	56.8F (1.6)	39.6E (1.1)	31.9E (1.2)
Semiarid area	Well-watered	51.6A (0.6)	65.5AB (1.0)	63.9CD (1.0)	47.6AB (0.9)	45.5A (1.1)
	Intermediately stressed	47.9BC (0.8)	63.7BC (0.7)	67.9A (0.7)	48.1AB (0.7)	39.6BC (0.8)
	Severely stressed	44.8DE (0.9)	58.0EF (1.4)	59.5EF (1.3)	41.7DE (1.1)	37.3CD (1.1)
Moist area	Well-watered	53.1A (0.7)	66.8A (0.8)	67.2AB (0.9)	49.5A (1.0)	46.0A (1.0)
	Intermediately stressed	49.0B (0.8)	63.9BC (0.8)	66.5ABC (0.7)	47.9AB (0.8)	41.7B (0.7)
	Severely stressed	46.2CD (1.0)	59.9DE (1.5)	62.0DE (1.3)	43.4CD (1.1)	40.6B (1.2)

Note: n = 24; different letters after data within a column indicate significant differences among treatments at the *P* < 0.05 level, nested ANOVA followed by Tukey tests.

The parasitism rate of *S*. *avenae* clones showed a tendency to decrease with increasing water-deficit extents in water treatments. The parasitism rate of 1^st^ instar nymphs for *S*. *avenae* clones from any area decreased significantly with increasing water-deficit levels (*F* = 63.25; df = 2, 207; *P* < 0.001); third instar nymphs of arid area clones had a lower parasitism rate under severely stressed conditions than under well-watered or intermediately stressed conditions (*F* = 33.86; df = 2, 207; *P* < 0.001); the parasitism rate of 4^th^ instar nymphs of arid area clones declined significantly with increasing water-deficit levels (*F* = 37.06; df = 2, 207; *P* < 0.001); and *S*. *avenae* adults of any area tended to have lower parasitism rates under higher levels of water-deficit (*F* = 52.64; df = 2, 207; *P* < 0.001).

### Developmental time of *A*. *gifuensis*

Under well-watered conditions, the developmental time of *A*. *gifuensis* on arid area clones of *S*. *avenae* was longer than that on semiarid or moist area clones for any developmental stage of the aphid (Table 4; 1^st^ instar: *F* = 33.60; df = 2, 207; *P* < 0.001; 2^nd^ instar: *F* = 38.58; df = 2, 207; *P* < 0.001; 3^rd^ instar: *F* = 36.58; df = 2, 207; *P* < 0.001; 4^th^ instar: *F* = 27.51; df = 2, 207; *P* < 0.001; adult: *F* = 32.61; df = 2, 207; *P* < 0.001). Under intermediately stressed conditions, the developmental time of *A*. *gifuensis* on *S*. *avenae* of all stages increased with increasing levels of water-deficit in source areas. A similar pattern was found under severely stressed conditions.

**Table 4:** Comparison of developmental times (SE) of *Aphidius gifuensis* on all developmental stages of *Sitobion avenae* clones from arid, semiarid and moist areas under three water treatments.

Source	Treatment	1^st^ instar	2^nd^ instar	3^rd^ instar	4^th^ instar	Adult
Arid area	Well-watered	10.9BC (0.2)	11.1B (0.2)	11.3B (0.2)	11.4B (0.2)	12.3BC (0.1)
	Intermediately stressed	11.4A (0.2)	11.9A (0.2)	12.0A (0.2)	11.5B (0.2)	12.7B (0.2)
	Severely stressed	11.6A (0.1)	11.7A (0.1)	11.9A (0.1)	12.0A (0.1)	13.2A (0.1)
Semiarid area	Well-watered	10.2DE (0.1)	10.4C (0.1)	10.6C (0.1)	10.8C (0.1)	11.8DE (0.1)
	Intermediately stressed	10.6CD (0.1)	11.0B (0.2)	11.3B (0.2)	10.9C (0.2)	12.0CD (0.1)
	Severely stressed	11.2AB (0.1)	11.6A (0.1)	11.6AB (0.1)	11.7AB (0.1)	12.4B (0.2)
Moist area	Well-watered	10.1E (0.2)	10.2C (0.1)	10.4C (0.1)	10.6CD (0.1)	11.4EF (0.2)
	Intermediately stressed	10.0E (0.2)	10.4C (0.2)	10.5C (0.1)	10.2D (0.2)	11.3F (0.2)
	Severely stressed	10.9BC (0.1)	11.0B (0.2)	11.3B (0.1)	11.5B (0.1)	12.5B (0.1)

Note: n = 24; different letters after data within a column indicate significant differences among treatments at the *P* < 0.05 level, nested ANOVA followed by Tukey tests.

When using 1^st^ to 3^rd^ instar nymphs of *S*. *avenae* (1^st^ instar: *F* = 25.54; df = 2, 207; *P* < 0.001; 2^nd^ instar: *F* = 27.21; df = 2, 207; *P* < 0.001; 3^rd^ instar: *F* = 27.00; df = 2, 207; *P* < 0.001), the developmental rate of *A*. *gifuensis* was higher under well-watered conditions than under intermediately or severely stressed conditions. For 4^th^ instar nymphs (*F* = 34.78; df = 2, 207; *P* < 0.001) and adults (*F* = 28.61; df = 2, 207; *P* < 0.001) of *S*. *avenae* clones from arid areas, the developmental time of *A*. *gifuensis* was longer under severely stressed conditions than under well-watered or intermediately stressed conditions. A similar pattern was found for semiarid or moist area clones. The parasitoid’s development on 2^nd^ and 3^rd^ instar nymphs of semiarid area clones was slower under the intermediately stressed treatment than under the well-watered treatment.

## Discussion

In the context of global warming, increasing intensity and frequency of drought events can have significant impacts on herbivores like aphids in agricultural systems. In our study, the developmental time of *S*. *avenae* clones from source areas (i.e., moist, semiarid and arid) tended to be prolonged on wheat plants under increasing levels of water-deficit (i.e., well-watered, intermediately stressed, and severely stressed). Arid area clones of *S*. *avenae* had smaller body sizes than moist area clones under well-watered conditions, and *S*. *avenae* clones from all areas had declining body sizes on wheat plants growing under increasing water-deficit levels. However, it has been reported that levels of proteins and amino acids can be increased in leaf tissues of plants under drought, which may result in faster development and bigger body size of aphids [13,41]. One explanation for our results is that drought can enhance mesophyll or phloem resistance (probably due to changes in phloem sap viscosity and solute concentrations), and thus increase the difficulty for aphids to get substantial amount of nutrients [42–43]. In addition, alterations in the water potential of host plants under drought may undermine the ability of aphids to consume xylem sap [44]. These changes of 1^st^ trophic level could have caused the abovementioned bottom-up effects on the 2^nd^ trophic level aphid. Our previous studies showed that *S*. *avenae* clones from arid areas had a relatively low fecundity, and that their adaptation potential was positively correlated with their body size [26–27]. Thus, *S*. *avenae* clones under drought can have not only lower fitness, but also lower adaptation potential, showing negative effects of water-deficit stressed plants on this aphid.

The survival and fitness of *S*. *avenae* under drought can also be affected by top-down effects of natural enemies. In our study, in terms of its plant contact behavior, the parasitoid (*A*. *gifuensis*) preferred *S*. *avenae* clones of moist areas over those of arid areas under all water treatments except severe water stress. Similarly, the number of attacks (/30 min) on moist area aphid clones by *A*. *gifuensis* was much higher than that on arid area clones under all three water treatments. Since semiochemicals from aphids and their habitats can provide cues for parasitoids to locate target prey [45–46], certain cues from moist area clones of *S*. *avenae* might be more concentrated (or attractive) for the parasitoid (*A*. *gefuensis*) than those from arid area clones. In this study, the parasitoid also preferred to attack *S*. *avenae* clones under well-watered conditions over those under water-deficit stress. This suggests that plants carrying *S*. *avenae* individuals may emit more attractive semiochemicals under well-watered conditions than those under water-deficit conditions [47]. Subsequently, arid area clones of *S*. *avenae* showed lower parasitism rates by *A*. *gifuensis* than moist area clones, and the parasitism rate for any developmental stage of *S*. *avenae* clones from different areas tended to decrease with increasing water-deficit extents in water treatments. An alternative explanation for lower parasitism of *S*. *avenae* clones under water-deficit conditions is that *A*. *gifuensis* can prefer to choose larger prey individuals in order to maximize its fitness. This makes sense since large prey can represent quality hosts (larger body size means more nutrition and space) for the parasitoid [48–49]. In addition, lower parasitism rates of *A*. *gifuensis* under drought can be related to aphid resistance behaviors (e.g., kicking, backing and dropping). In this study, relative to arid area clones, moist area clones of *S*. *avenae* had a higher frequency of backing under severely stressed conditions only, but a higher frequency of kicking under well-watered conditions only. The water-deficit level dependent pattern of *S*. *avenae*’s resistance against parasitoids could be important for the survival of this aphid under different drought conditions. Endosymbionts (e.g., *Hamiltonella defensa*) could also be important in successful defense against *A*. *gifuensis* for *S*. *avenae* clones under drought [50]. Further studies are needed to determine how endosymbionts of this aphid can contribute to the abovementioned differential performances of *S*. *avenae* clones confronting the parasitoid.

In addition to the abovementioned factors on the aphid side, the top-down effects of *A*. *gifuensis* under drought are highly dependent on the factors of the parasitoid side (e.g., development). In this study, the development of *A*. *gifuensis* on arid area clones of *S*. *avenae* (all developmental stages) was slower than that on moist area clones under all water treatments. Similarly, under intermediate water stress, *A*. *gifuensis*’s developmental rates on *S*. *avenae* of all stages decreased with increasing levels of water-deficit in source areas (i.e., moist, semiarid and arid). The developmental time of the parasitoid on all stages of *S*. *avenae* clones also tended to increase with increasing levels of water-deficit in water treatments. Such results suggest that the fitness of *A*. *gifuensis* could be decreased when preying on *S*. *avenae* clones of any origin under water-deficit conditions. This could further compromise its preying effects on *S*. *avenae* under increasing drought conditions. Therefore, the top-down effects of natural enemies (e.g., *A*. *gifuensis*), but not the bottom-up effects of the water-stressed first trophic level, can partly explain the phenomenon that increasing events of *S*. *avenae* outbreaks on cereal crops coincide with the climate change trend in drought-inflicted northwestern China [51–52].

Overall, our results are consistent with the finding that drought conditions (or water-deficit stress) have significant impacts on insect pests and their natural enemies [5,53]. It is evident from our study that the development of *S*. *avenae* can be slowed and its body size can be reduced under drought, showing negative bottom-up effects of water-deficit stressed plants. However, the performance of the parasitoid (*A*. *gifuensis*) is also negatively affected by water-deficit stress, suggesting that the top-town effects via parasitoids can be compromised by drought. Thus, the water-deficit-stressed first trophic level can have indirect and negative impacts on the performance of the third trophic level parasitoids. This suggests that parasitoids can be more sensitive to future warming scenarios than other trophic levels [5,53], and that their vulnerability can be potentially increased in future warming scenarios. The rapid adaptation of aphids to water-deficit stress, as well as wide-spread occurrences of their local adaptation, have been demonstrated in some studies [26,28–29,54–55]. This could push parasitoids of aphids to an increasingly vulnerable status in the face of the climate change through their interactions shown in this study. In order to enhance our understanding of how water-deficit stress affects tritrophic (e.g., plant-insect pest-natural enemy) interactions, further studies should be conducted to examine the ecological and evolutionary consequences in field conditions as well as the underlying mechanisms from a perspective of molecular ecology.
